# Immune cells‐derived exosomes: A promising strategy for COVID‐19 treatment

**DOI:** 10.1002/ctd2.138

**Published:** 2022-10-10

**Authors:** Zichen Zhang, Jiangang Long, Lingjie Meng, Zhiwei Yang, Shengli Zhang, Lei Zhang

**Affiliations:** ^1^ MOE Key Laboratory for Nonequilibrium Synthesis and Modulation of Condensed Matter, School of Physics Xi'an Jiaotong University Xi'an China; ^2^ School of Life Science and Technology Xi'an Jiaotong University Xi'an China; ^3^ School of Chemistry Xi'an Jiaotong University Xi'an China; ^4^ Instrumental Analysis Center Xi'an Jiaotong University Xi'an China

**Keywords:** COVID‐19, immune cells‐derived exosomes, immune modulation, immune responses

Coronavirus disease 2019 (COVID‐19) has threatened the lives and health of billions of people since it swept across the world in December 2019.^1^ It causes pulmonary edema, dyspnea and lung failure in severe cases.^2^ The speedy infectivity of the virus limits the movement of people, causing economic stagnation, collapse of infrastructure and severe global shock.^3^ Frequent mutations of severe acute respiratory syndrome coronavirus 2 (SARS‐CoV‐2) make the treatment and prevention of COVID‐19 a genuine challenge.^4^ Fortunately, exosomes (Exos) derived from immune cells have the trend to expedite the exhaustion of immune cells, as well as regulate viral transmission, replication, and mutations (Figure 1). Thereby, the modulation of immune function by Exos is promising to the treatment of COVID‐19.

**FIGURE 1:**
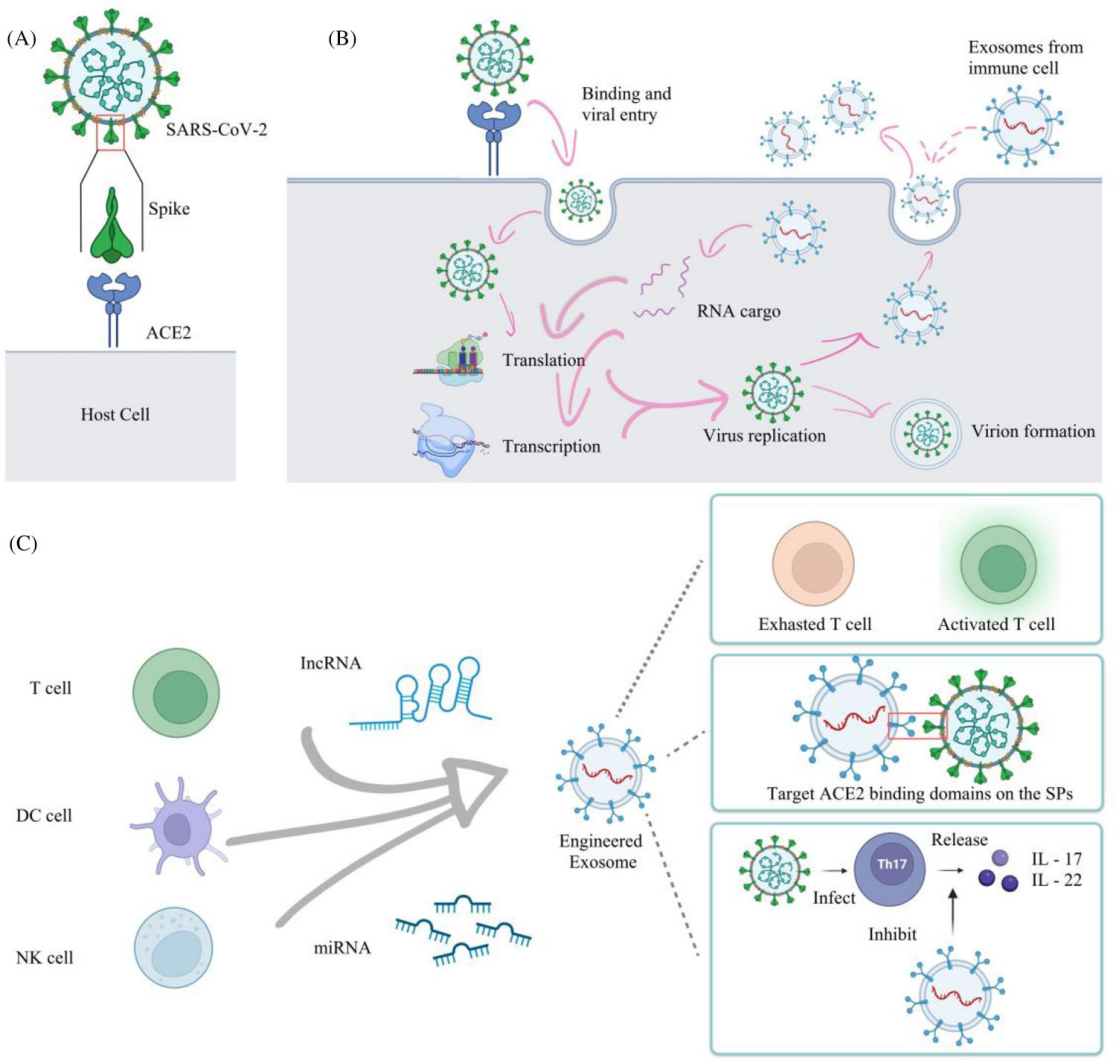
(A) Severe acute respiratory syndrome coronavirus 2 (SARS‐CoV‐2) targets host angiotensin‐converting enzyme 2 (ACE2) receptor by spike proteins (SPs). (B) The process of regulating viral transcription and replication. (C) Three strategies of engineered Exos in treating COVID‐19

At present, there are a lot of researches and reviews on Exos and extracellular vesicles against COVID‐19, while most of them focus on the role and possibility of treatment in specific sources,^2^, ^5^, ^6^, ^7^ without the whole research progress in the era of COVID‐19 outbreak.^8^ A recent review published in *Clinical and Translational Medicine* presents important insights into current immunopathology and the applications of immune cell‐derived Exos for the immunotherapy of COVID‐19.^9^ Exos derived from immune cells could trigger novel anti‐SARS‐CoV‐2 immune responses, which are effective in reducing the inflammatory response. In addition, Exos inhibits the proliferation and activity of T helper cell 17 (Th17), which plays an important role in inducing respiratory failure through the delivery of regulatory miRNAs. These features arouse increasing attention on Exos with an enormous potential efficacy in the immunotherapy clinical treatment of COVID‐19; however the underlying mechanisms remain to be elucidated.

Due to the ribosomal proteins (RPs) capacity of blocking the SARS‐CoV‐2 replication and stimulating immune targeting of infected cells,^10^ there are good reasons to believe that Exos should be directed towards targeting RPs to inhibit the replication of viral genome.^11^ A novel strategy has been thus proposed with loading miRNAs, which bind to the SARS‐CoV‐2 genome and inhibit post‐transcriptional expressions on Exos derived from tolerogenic dendritic cells (tolExos).^12^ Alternatively, mesenchymal stem cells (MSCs) have become the preferred candidates for the protection of alveolar epithelial cells benefitting from the capacity to regulate immune responses and reduce inflammation.^13^ MSCs‐derived Exos inhibits HIV replication through inducing RNA/DNA methylation suggesting that epigenetically modified Exos could contribute to reducing viral replication and may be an ideal intervention to treat COVID‐19.^14^ Besides, MSC‐derived tolExos and tolExos loaded fibroblast growth factor 7 reduce the inflammation symptoms and promote lung repair.^15^

Exos derived from B cells and T cells has played important roles in AIDS treatment by activating T cell function and stimulating B cell responses.^16^ The huge differences in gene expression of Exos derived from exhausted and non‐exhausted CD8^+^ T cells were confirmed by using microarray analysisimmediately,^17^ which further supported the potentiality of immune cells‐derived exosomes for the COVID‐19 treatment. These differences were mainly reflected in the expression of lncRNAs. Thereby we have reason to believe that employing Exos harboring active lncRNAs that could be a promising strategy for restoring functional T cells in COVID‐19.^18^

Compared to antibody therapy and small molecule, the ability to home at its origin tissue, cell processes control through mediating multifunction and simple differentiation and labeling give Exos numerous advantages in the treatment of COVID‐19.^9^ While there is no definite theoretical basis for the immune modulation mechanisms driven by Exos, especially corresponding dynamic structure alterations involved in the interactions among Exos, virions, and host cells. With the development of super‐resolution microscopy (SRM), fluorescence images of the interaction between Exos and SARS‐CoV‐2 beyond the diffraction limit have been possible, and the detailed structures related to Exos can be benefited from cryo‐electron microscopy (cryo‐EM). Molecular dynamics simulation could overcome the scale gap between SRM and cryo‐EM, and the combination of the SRM, cryo‐EM and MD simulation will be an effective solution for the research of the dynamic structure and the interaction between Exos and targets. Because of the dynamic nature of the physiological process, mechanism analysis based on the whole process of dynamic evolution has become a very important link in the treatment of COVID‐19, which will become the foundation of Exos directional modification for the defense and treatment methods.

## AUTHOR CONTRIBUTIONS

Mr. Zichen Zhang, Dr. Zhiwei Yang and Dr. Lei Zhang contributed to the preparation and collection of original literatures and figures and the writing and editing of manuscript. Dr. Zhiwei Yang, Dr. Jiangang Long, Dr. Lingjie Meng and Dr. Shengli Zhang were responsible for the structural designs, scientific quality and writing.

## FUNDING INFORMATION

This research was supported by the National Natural Science Foundation of China (No. 11774279, 11774280) and the National Science Fund for Outstanding Young Scholars (No. 11922410).

## CONFLICT OF INTEREST

The authors declare no conflict of interest. The paper was handled by editors and has undergone a rigorous peer‐review process. Dr. Zhiwei Yang was not involved in the journal's review of/or decisions related to this manuscript.

## ETHICAL APPROVAL

Not applicable.

## Data Availability

Data sharing is not applicable to this article as no new data were created or analyzed in this study.
